# Incidence, predictors, and indications of permanent pacemaker implantation following orthotopic heart transplantation: A single-center experience

**DOI:** 10.1016/j.jhlto.2025.100276

**Published:** 2025-05-02

**Authors:** Iriagbonse Rotimi Asemota, Siddharth Das, Michelle Nsahlai, Edward Graviss, Duc T. Nguyen, Paul A. Schurmann, Rayan Yousefzai

**Affiliations:** a Houston Methodist Hospital, Houston, Texas; b Ascension St Vincent, Indianapolis, Indiana; c Department of Pediatrics, Baylor College of Medicine, Houston, Texas

**Keywords:** permanent pacemaker implantation, orthotopic heart transplant, arrythmia, heart transplant outcome, pacing

## Abstract

In the modern era of orthotopic heart transplantation (OHT) with bicaval anastomosis, there is a paucity of data on permanent pacemaker implantation (PPI) in this population. This is a retrospective study describing the incidence, indication, and predictors in patients who underwent PPI after OHT. Using the transplant database from our institution between January 2016 and December 2022, 370 patients who had de-novo OHT were identified. The incidence of PPI post-OHT was 8.1% (*n* = 30 patients). The average time from OHT to PPI was 30.5 days. The most common indication for PPI was sinus node dysfunction, accounting for 83% of cases. Regarding predictors for PPI, multivariate analysis identified donor age as an independent predictor (hazard ratio 1.05, 95% confidence interval 1.01-1.09, *p* = 0.02). However, factors, including recipient age, ischemic time, sex, underlying cardiomyopathy, body mass index, mechanical support, pretransplant length of stay, and post-OHT length of stay, did not show significant predictive value.

## Background

Orthotopic heart transplantation (OHT) remains an effective treatment for end-stage heart failure, with a median survival of about 13 years.^1^ Bradycardia may occur post-OHT and sometimes requires permanent pacemaker implantation (PPI), as outlined in the 2018 American Heart Association/American College of Cardiology/Heart Rhythm Society (AHA/ACC/HRS) Guidelines.^2^ PPI rates have declined with the shift from biatrial to bicaval anastomosis, which reduces atrial manipulation and sinus node injury.^3^ However, data on PPI incidence, indications, and predictors remain limited and vary across institutions. The United Network for Organ Sharing (UNOS) database (2000-2021) reported a 2.9% PPI rate among 49,529 recipients, without specifying bicaval usage.^4^ This retrospective study evaluates PPI incidence, indications, and predictors using contemporary data from a high-volume center employing a 100% bicaval approach.

## Study population and methods

We retrospectively reviewed 370 heart transplant recipients from January 2016 to December 2022. Patient demographics and characteristics were compared between those who required PPI post-OHT and those who did not. The primary outcome was PPI incidence; secondary outcomes included predictors, indications, pacing use, and arrhythmias based on device interrogations. Categorical variables were summarized as frequencies/proportions, and continuous variables as medians with interquartile range (IQR). Group differences were assessed using chi-square/Fisher’s exact tests and Wilcoxon rank-sum tests. Kaplan-Meier curves estimated cumulative PPI incidence; Cox regression identified associated factors. Model performance was evaluated via C-statistic. Analyses used Stata 17.0 (StataCorp), with significance set at *p* < 0.05. Institutional Review Board (IRB) approval was obtained.

## Results

**Figure 1:**
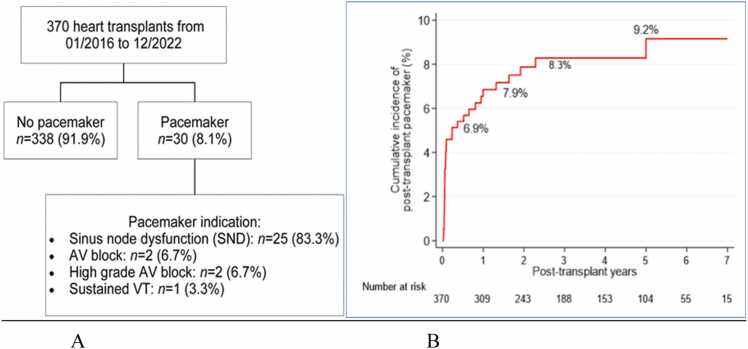
(A) Incidence and indications for PPM implantation post heart transplant. (B) Cumulative incidence of permanent pacemaker implantation post heart transplant. AV block, atrioventricular block, SND, sinus node dysfunction; VT, ventricular tachycardia.

Demographics and clinical characteristics of patients stratified based on those who received PPI and those who did not are shown in Table 1. A total of 370 OHTs were included in the study, 30 patients (8.1%) required PPI (Figure 1A). Preoperative use of amiodarone among patients with PPI after OHT was 48%. Our institutional practice involves the intraoperative placement of temporary epicardial pacing wires, which are subsequently removed upon confirmation of sinus node activity and adequate atrioventricular (AV) node conduction. This is followed by a monitoring period of approximately 2 to 3 weeks depending on the recovery, during which beta-agonists or other adrenergic agents are administered as needed. Permanent pacemaker (PPM) implantation is then considered for patients with persistent sinus node dysfunction (SND), chronotropic incompetence, or AV block. The most common indication for PPI after OHT was SND (83.3%), followed by AV block (6.7%) and 1 patient who had ventricular tachycardia required PPI as part of the defibrillator placement (Figure 1A). Figure 1B displays the cumulative incidence of PPM insertion post-OHT. On average, the time elapsed from OHT to PPM was 30.5 days. There was a similar post-transplant length of stay (22.2 vs 21.1 days, *p* = 0.9) and ischemic time (213 vs 216 minutes, *p* = 0.46) between the PPI group and the non-PPI group.

**Table 1:** Patient Characteristics

Variables	Total (*n* = 370)	No pacemaker (*n* = 338)	Pacemaker (*n* = 30)	*p*-value
*Recipient characteristics*				
Age (years), median (IQR)	58.6 (51.4, 64.0)	58.8 (52.0, 64.0)	57.3 (45.0, 65.0)	0.32
Gender				
Female Male	108 (29.2) 262 (70.8)	101 (29.7) 239 (70.3)	7 (23.3) 23 (76.7)	0.46
BMI (kg/m^2^), median (IQR)	26.6 (23.4, 30.0)	26.6 (23.4, 30.3)	25.8 (23.4, 27.7)	0.17
Cardiomyopathy				0.52
Ischemic Non ischemic Infiltrative Congenital heart disease Genetic Hypertrophic Pulmonary arterial HTN	119 (32.2) 182 (49.2) 40 (10.8) 14 (3.8) 4 (1.1) 7 (1.9) 4 (1.1)	112 (32.9) 164 (48.2) 35 (10.3) 14 (4.1) 4 (1.2) 7 (2.1) 4 (1.2)	7 (23.3) 18 (60.0) 5 (16.7) 0 (0.0) 0 (0.0) 0 (0.0) 0 (0.0)	
Type of transplant				0.64
Heart Heart/kidney Heart/liver Heart/lung Heart/kidney/liver	243 (65.7) 74 (20.0) 28 (7.6) 20 (5.4) 5 (1.4)	221 (65.0) 68 (20.0) 26 (7.6) 20 (5.9) 5 (1.5)	22 (73.3) 6 (20.0) 2 (6.7) 0 (0.0) 0 (0.0)	
Mechanical support				0.15
None IABP Impella LVAD LVAD + ProtekDuo TAH ECMO	45 (12.2) 225 (60.8) 33 (8.9) 57 (15.4) 1 (0.3) 2 (0.5) 7 (1.9)	41 (12.1) 211 (62.1) 26 (7.6) 53 (15.6) 1 (0.3) 2 (0.6) 6 (1.8)	4 (13.3) 14 (46.7) 7 (23.3) 4 (13.3) 0 (0.0) 0 (0.0) 1 (3.3)	
LOS pretransplant (days), median (IQR)	34.5 (12.4, 60.6)	34.5 (12.3, 60.6)	27.7 (13.3, 54.3)	0.79
LOS post-transplant (days), median (IQR)	21.2 (16.0, 31.0)	21.1 (16.0, 31.6)	22.2 (16.4, 27.9)	0.9
Total LOS (days), median (IQR)	58.4 (35.1, 89.8)	59.7 (35.1, 90.0)	55.1 (36.3, 84.3)	0.74
				
*Donor characteristic*				
Donor age (years), median (IQR)	28.0 (22.0, 36.0)	28.0 (22.0, 35.0)	34.5 (25.5, 41.5)	0.04
Ischemic time (minutes), median (IQR)	216.0 (172.0, 243.0)	216.0 (175.0, 243.0)	13.0 (140.0, 245.0	0.46

Abbreviations: BMI, body mass index; ECMO, extracorporeal membrane oxygenation; HTN, hypertension; IABP, intra-aortic balloon pump; IQR, interquartile range; LOS, length of stay; LVAD, left ventricular assist device; TAH, total artificial heart.

Values are in numbers (%) for categorical variables and median (IQR) for continuous variables.

Most pacemakers implanted were dual-chamber right atrium/right ventricle (RA/RV) devices (73.3%), followed by single-lead RA (20%), dual-chamber (RA/His) (3.3%), and 3-lead right atrium/right ventricle/left ventricle (RA/RV/LV) devices (3.3%). As regards pacing utilization, the interrogation report showed that the average atrial pacemaker rate over 2 years post-transplant was 20% ± 24, while the average ventricular pacemaker rate was 2% ± 6. Other arrhythmias detected on pacemaker interrogation included atrial flutter (4%), atrial fibrillation (12%), supraventricular tachycardia (4%), nonsustained ventricular tachycardia (32%), and ventricular tachycardia (4%). Regarding predictors of PPM implantation, after conducting multivariate analysis, donor age emerged as a positive predictor (hazard ratio 1.05, 95% confidence interval 1.01-1.09, *p* = 0.02) as seen in Table 2. However, other factors such as recipient age, ischemic time, sex, underlying cardiomyopathy, body mass index, mechanical support before transplant, pretransplant length of stay, and post-OHT length of stay were not predictive of need for PPI.

**Table 2:** Factors Associated With Having Pacemaker After Transplantation, Univariable and Multivariable Cox Regression

Variables	Univariable		Multivariable	
	HR (95% CI)	*p*-value	HR (95% CI)	*p*-value
*Recipient characteristics*				
Age (years), median (IQR)	0.98 (0.95, 1.01)	0.15	0.98 (0.95, 1.01)	0.17
Gender	(Reference)	0.45	–	–
Female				
Male	1.38 (0.59, 3.22)		–	–
BMI (kg/m^2^), median (IQR)	0.95 (0.88, 1.03)	0.21	–	–
Cardiomyopathy	(Reference)	0.21	–	–
Ischemic				
Non ischemic	1.75 (0.73, 4.19)		–	–
Infiltrative	2.29 (0.73, 7.22)	0.16	–	–
Congenital heart disease	–	–	–	
Genetic	–	–	–	–
Hypertrophic	–	–	–	–
Pulmonary arterial hypertension	–	–	–	–
Cardiomyopathy	(Reference)	0.21	(Reference)	0.23
Ischemic				
Non ischemic	1.75 (0.73, 4.19)		1.80 (0.69, 4.67)	
Other	1.32 (0.42, 4.16)	0.64	1.31 (0.40, 4.35)	0.66
Type of transplant	(Reference)	0.92	–	–
Heart				
Heart/kidney	0.95 (0.39, 2.35)		–	–
Heart/liver	0.81 (0.19, 3.43)	0.77	–	–
Heart/lung	–	–	–	–
Heart/kidney/liver	–	–	–	–
Mechanical support	(Reference)	0.51	–	–
None				
IABP	0.69 (0.23, 2.09)		–	–
Impella	2.70 (0.79, 9.24)	0.11	–	–
LVAD	0.73 (0.18, 2.93)	0.66	–	–
LVAD + ProtekDuo	–	–	–	–
TAH	–	–	–	–
ECMO	1.87 (0.21, 16.78	0.57	–	–
LOS pretransplant (days), median (IQR)	1.00 (0.99, 1.01)	0.85	–	–
LOS post-transplant (days), median (IQR)	1.00 (0.98, 1.02)	0.68	–	–
Total LOS (days), median (IQR)	1.00 (0.99, 1.01)	0.76	–	–
				
*Donor characteristic*				
Donor age (years), median (IQR)	1.05 (1.00, 1.09)	0.03	1.05 (1.01, 1.09)	0.02
Ischemic time (minutes), median (IQR)	1.00 (0.99, 1.00)	0.31	–	–

Abbreviations: BMI, body mass index; CI, confidence interval; ECMO, extracorporeal membrane oxygenation; HR, hazard ratio; HTN, hypertension; IABP, intra-aortic balloon pump; IQR, interquartile range; LVAD, left ventricular assist device; TAH; total artificial heart.

C-statistic = 0.67. *n* = 333, number of patients having complete data for all variables used in the multivariable Cox model.

## Discussion

Our center’s PPI rate after 100% bicaval anastomotic OHT was 8.1%, which is consistent with the trend of lower PPM rates in OHT recipients compared to prior studies with biatrial technique with PPI rates from 11% to 14%.^4^, ^5^ Our study also explores the complex interplay between surgical technique (bicaval anastomosis), donor and recipient characteristics, and post-transplant arrhythmias and PPM use. To our knowledge, this level of detail has not been examined within the same cohort in prior studies. Prior studies have combined biatrial and bicaval approach with historical transplant cohort dating back to 1980s with decades of OHT collected.^6^ In the few studies with 100% bicaval technique, PPI rates after OHT vary from as low as 3.8% to as high as 20%.^7^, ^8^ In contrast, the largest UNOS database report in PPM implantation rate of 2.3%, however, did not report the number of bicaval surgical approaches used.^3^ The variance in PPI rate post-OHT may be explained by the difference in institutional policy as regards the timing of waiting and observation period before PPI.

SND remains the most common indication for PPI after OHT in our cohort which is consistent with prior studies. Donor SND is the most common cause of bradyarrhythmia reported after transplantation.^9^ Older donor heart is more likely to have age-related degeneration of the cardiac conduction system with fibrosis and degeneration of the sinoatrial and AV node hence predispose to persistent bradyarrhythmia. Zieroth et al showed that with every 5-year increase in donor age, the risk of post-transplant PPM increased 1.2-fold.^10^ The average time to PPM placement in our study was 30 days. There may be a need for further research to guide and optimize the watch-and-wait approach to reduce the PPI after OHT. The pacemaker utilization rate in these patients within the first 2 years of implantation was atrial pacing usage of 20% and ventricular pacing usage of 2%, respectively. The programming mode of Atrial paced, atrial sensed, atrial inhibited-dual pacing, dual sensing, dual response (AAI-DDD) automatic mode switch algorithm was used for all PPM to minimize the rate of ventricular pacing and minimize the possibility of pacing-induced cardiomyopathy.

Our study has several limitations. This single-center retrospective study is limited by potential confounding and sample size, but benefits from a uniform 100% bicaval technique, offering insights into modern recipient, donor, and predictor factors.

## Conclusion

The rate and indication for PPI after OHT vary widely among centers. At our center, the bicaval OHT PPI rate was approximately 8%, primarily due to SND. There is a need for standardization of pacemaker utilization and indications in post-OHT patients across centers.

## CRediT authorship contribution statement

**Iriagbonse Rotimi Asemota:** Data acquisition, data interpretation, manuscript writing/review, final approval. **Siddharth Das:** Data acquisition, analysis and interpretation, final approval. **Michelle Nsahlai:** Study design, data acquisition, analysis and interpretation, final approval. **Duc T. Nguyen:** Data analysis and interpretation, final approval. **Edward Graviss:** Data analysis and interpretation, final approval. **Paul A. Schurmann:** Study design, analysis and interpretation, final approval. **Rayan Yousefzai:** Study design, manuscript writing/review, supervision, data analysis and interpretation, final approval.

## Disclosure statement

The authors declare that they have no known competing financial interests or personal relationships that could have appeared to influence the work reported in this paper.

Funding and Acknowledgments: None.
